# Potential Genes Related to Levofloxacin Resistance in *Mycobacterium tuberculosis* Based on Transcriptome and Methylome Overlap Analysis

**DOI:** 10.1007/s00239-019-09926-z

**Published:** 2020-01-09

**Authors:** Hai-cheng Li, Hui-xin Guo, Tao Chen, Wei Wang, Zhu-hua Wu, Liang Chen, Hui-zhong Wu, Gao-po Xu, Xun-xun Chen, Lin Zhou

**Affiliations:** 1 Centre for Tuberculosis Control of Guangdong Province, 485 West Huangpu Avenue, Guangzhou, 510630 China; 2 The Third Affiliated Hospital, Sun Yat-Sen University, No. 600 Tianhe Road, Guangzhou, 510630 China; 3 The Forth People’s Hospital of Foshan, Foshan, 528000 China

**Keywords:** *Mycobacterium tuberculosis*, Transcriptome, Methylome, Differentially expressed genes, Differentially methylated genes, Levofloxacin resistance

## Abstract

Drug-resistant *Mycobacterium tuberculosis* (*M. tuberculosis*) has become an increasingly serious public health problem and has complicated tuberculosis (TB) treatment. Levofloxacin (LOF) is an ideal anti-tuberculosis drug in clinical applications. However, the detailed molecular mechanisms of LOF-resistant *M. tuberculosis* in TB treatment have not been revealed. Our study performed transcriptome and methylome sequencing to investigate the potential biological characteristics of LOF resistance in *M. tuberculosis* H37Rv. In the transcriptome analysis, 953 differentially expressed genes (DEGs) were identified; 514 and 439 DEGs were significantly downregulated and upregulated in the LOF-resistant group and control group, respectively. The KEGG pathway analysis revealed that 97 pathways were enriched in this study. In the methylome analysis, 239 differentially methylated genes (DMGs) were identified; 150 and 89 DMGs were hypomethylated and hypermethylated in the LOF-resistant group and control group, respectively. The KEGG pathway analysis revealed that 74 pathways were enriched in this study. The overlap study suggested that 25 genes were obtained. It was notable that nine genes expressed downregulated mRNA and upregulated methylated levels, including *pgi*, *fadE4*, *php*, *cyp132*, *pckA*, *rpmB1*, *pfkB*, *acg*, and *ctpF*, especially *cyp132*, *pckA*, and *pfkB*, which were vital in LOF-resistant *M. tuberculosis* H37Rv. The overlapping genes between transcriptome and methylome could be essential for studying the molecular mechanisms of LOF-resistant *M. tuberculosis* H37Rv. These results may provide informative evidence for TB treatment with LOF.

**Electronic supplementary material:**

The online version of this article (10.1007/s00239-019-09926-z) contains supplementary material, which is available to authorized users.

## Introduction

Tuberculosis (TB) is an infectious disease usually caused by *Mycobacterium tuberculosis* (*M. tuberculosis*) infection (Zaman 2010). According to a World Health Organization (WHO) report, there were 10 million new cases of TB and 1.3 million deaths as a result of TB in 2017. In India, China, and Indonesia, the total number of people with TB accounts for 44% of the cases worldwide. In China, about 889,000 TB cases were reported, accounting for 9% of the cases worldwide (WHO 2018). Therefore, the prevention of TB remains an urgent task. In recent years, the global TB epidemic has declined. Multidrug-resistant TB (MDR-TB) refers to TB that is resistant to two or more anti-TB drugs, such as rifampicin and isoniazid (Seung et al. 2015). The clinical abuse of anti-TB drugs has led to an increasing number of drug-resistant strains. Moreover, the failure rate of MDR-TB treatment is significantly higher than that at which susceptible bacteria are infected with TB (Ormerod 2005). As such, the development and testing of new drugs are direly needed to enable more timely and effective treatment of MDR-TB.

Levofloxacin (LOF) is a third-generation fluorinated quinolone antibiotic that is an optically active L-isomer of the racemate ofloxacin (Moorthy et al. 2008). LOF plays an important role in inhibiting the activity of the *M. tuberculosis* gyrase, which severely blocks the replication process of DNA. Therefore, this drug could effectively promote DNA degradation and kill bacteria in a short period time (Collin et al. 2011). The bactericidal activity of this molecule against intracellular and extracellular *M. tuberculosis* could be more than twice that of ofloxacin (McGregor et al. 2008). In recent years, it has been clinically proposed that LOF can directly inhibit the metabolism of Mycolic acid in mycobacteria (Ghimire et al. 2016). Thus, it plays a therapeutic role in treating TB. In clinical practice, LOF is an ideal anti-TB drug without any cross-resistance in combination with other clinically applied anti-TB drugs (Richeldi et al. 2002). However, evidence of the detailed molecular mechanisms of LOF resistance in TB is still insufficient.

The main process of DNA methylation is the conversion of the 5′-terminal cytosine of CpG dinucleotide to a 5ʹ-methylcytosine due to the activity of DNA methyltransferase and a methylated CpG-binding protein (Lister et al. 2009). There is evidence that DNA methylation modifications are closely associated with a variety of diseases (Robertson 2005), including tumorigenesis, infectious diseases, autoimmune diseases, chronic diseases, and blood diseases (Ramos et al. 2011; Saleem et al. 2015; Zhuang et al. 2012). Moreover, previous studies have highlighted occurrence of DNA methylation remodeling in dendritic cells upon infection with *M. tuberculosis* (Pacis et al. 2019, 2015). In addition, researchers have focused on the relationship between drug resistance and the *M. tuberculosis* complex (MTC) (Madsen et al. 2005; Phunpruch et al. 2013). For example, Phunpruch et al. suggested that the methylation pattern in *Mycobacterium smegmatis* (*M. smegmatis*) could be used to understand the resistance toward lincosamides and erythromycin (Phunpruch et al. 2013). Therefore, we speculate that DNA methylation may be closely related to LOF resistance in the *M. tuberculosis* H37Rv strain.

In this study, we have employed LOF to study the drug resistance in the *M. tuberculosis* H37Rv strain. The transcriptomes and methylomes of the LOF-resistant *M. tuberculosis* H37Rv strain and the wild-type *M. tuberculosis* H37Rv strain were studied using a high-throughput sequencing method. Differentially expressed genes (DEGs) and methylated genes (DMGs) have been investigated. KEGG pathway analysis has revealed the important signal pathways related to LOF resistance. In addition, a molecular regulation network has been constructed based on the DEGs and DMGs. The results provide useful information regarding LOF resistance in *M. tuberculosis* H37Rv.

## Materials and Methods

### Sample Treatment

In this study, the wild-type *M. tuberculosis* H37Rv strain was obtained from the Sample Bank of the Reference Laboratory of Guangdong Province and preserved in Löwenstein–Jensen (LJ) medium (Thermo Fisher Scientific, USA) at 37 °C for 4 weeks. Subsequently, a monoclone was chosen. The monoclonal agglomerate was dispersed and diluted to 1 Mech turbidity using a bacterial ultrasonic dispersion counter. The prepared bacterial solution was inoculated with LJ medium for amplification. This generation of strain was designated as the primary G0. The WHO criteria (Guidelines for surveillance of drug resistance in tuberculosis. WHO Geneva/IUATLD Paris. International Union Against Tuberculosis and Lung Disease 1998) for LOF-resistant strains suggested that *M. tuberculosis* H37Rv strains could survive in a medium containing LOF at 2.0 μg/mL. Therefore, the G0 strain was further cultured in LJ medium at 37 °C for 4 weeks with 2.0 μg/mL LOF (Sigma-Aldrich, USA). The drug-resistant strain was named the G1 generation. The same steps were repeated until G4 strains obtained, which satisfied the criteria provided by the WHO. Subsequently, three more generations of the drug-resistant G4 strains and the wild-type *M. tuberculosis* H37Rv strain were cultured under identical conditions (LJ medium at 37 °C for 4 weeks per generation). LOF-resistant G7 strains and the wild-type *M. tuberculosis* H37Rv strain were designated as the LOF-resistant group and control group, respectively. Bacteria in both groups were stored at − 80 °C for further DNA and RNA extraction.

### Transcriptome

The total RNAs of bacteria in the LOF-resistant and control groups were extracted using a MiniBEST Universal RNA Extraction Kit (Takara, Japan). Purity and concentration were determined using NanoDrop ND1000 (FisherScientific, USA). DNase I (Epicenter, USA) was used to remove DNA contamination. Ribosomal RNA was removed using an Epicenter Ribo-zero™ rRNA Removal Kit (Epicenter, USA). Linear RNA was fragmented into 180 bp using the ultrasonic method (Covaris M220, USA). After fragmentation and random priming, reverse transcription was carried out using a PrimeScript™ II Strand cDNA Synthesis Kit (Takara, Japan). An NEBNext® Ultra™ Directional RNA Library Prep Kit for Illumina® (NEB,USA) was used according to the manufacturer’s protocol. After the end repair, blunting, and adenylation of 3ʹ ends of cDNA fragments, an adaptor was ligated to each cDNA end. Subsequently, size selection (approximately 400 bp) was carried out using an AMPure XP system (Beckman, USA). Then, adaptor-ligated cDNA was amplified with universal PCR primers. The final library products were purified and assessed using an Agilent Bioanalyzer 2100 system (Agilent, USA). The clustering of the index-coded sample was performed using a cBot Cluster Generation System (Illumina, USA). The libraries were sequenced on an Illumina HiSeq 2500 platform with the 150-bp paired-end sequencing strategy, and the raw data were finally obtained. Subsequently, high-quality reads were obtained after the removal of low-quality reads (sequences containing the linker, > 10% N bases, or > 50% bases with a < 10 mass value). The aligned reads were obtained by mapping high-quality reads in the *M. tuberculosis* H37Rv genome (https://www.ncbi.nlm.nih.gov/genome/166?genome_assembly_id=159857) using HISAT (version 0.1.6, -phred33 -p 3 -x * -known-splicesite-infile *). The aligned reads were further mapped onto the gene exon region via HTseq (version 0.6.1, -count -f bam -r pos -s no -i gene_name). Finally, the mapped reads were used for differential expression analysis via DESeq (version 2) combined with R (version 3.1) under default parameters (Love et al. 2014).

### Methylome

DNA of 1 × 10^6^ bacteria cells in the LOF-resistant and control groups were obtained using a QIAamp DNA Mini Kit (Qiagen, USA). 100U MspI (NEB, USA) was used to digest the 5 μg genomic DNA from the two groups for 16 h at 37 °C. An Illumina sequencing library was constructed using a paired-end sequencing strategy. In brief, library preparation includes three main steps: end repair, “A” base addition, and methylated-adapter ligation. The size distribution of the library was examined with 2% agarose gel. A MinElute PCR Purification Kit (QIAGEN, USA) was used to recover DNA by columns using a 20 µl elution buffer. Bisulfite conversion was performed using an EZ DNA Methylation-Gold Kit (ZYMO, USA). Bisulfite-treated products were amplified by a 50 µl PCR reaction system, which contained 10 µl-treated DNA, 1 µl 10 mM dNTP, 1 µl PCR primer A, 1 µl PCR primer B, 5 µl 10 × master mix buffer, 0.5 µl JumpStartTM Taq DNA polymerase, and 31.5 µl water. The PCR program was implemented as follows: 94 °C 1 min, 11 cycles (94 °C 0.5 min, 58 °C 0.5 min, and 72 °C 0.5 min), 72 °C 5 min. The PCR products were purified and recovered with a QIAamp DNA Mini Kit (Qiagen, USA) followed by sequencing with Illumina HiSeq2500 with the 150-bp paired-end sequencing strategy. BGI SOAPaligner version 2.01 with two mismatches for successful mapping was used to identify the strand specificity of DNA methylation (Li et al. 2009). CpG loci with significant differences between the two groups in DNA methylation have been identified through surrogate variable analysis (Leek and Storey 2007).

### Bioinformatics

A heat map of DEGs and DMGs was created using the Cluster 3.0 software (USA) (FDR ≤ 0.001 and |log_2_ ratio| ≥ 1). KEGG signaling pathway analysis was conducted using the KOBAS software (version 3.0, https://kobas.cbi.pku.edu.cn/). The protein–protein interaction network analysis was based on the Search Tool for the Retrieval of Interacting Genes (STRING) (version 11.0, https://string-db.org/).

### Statistics

Data were expressed as mean ± SD. Spearman’s linear regression analysis was applied to identify the correlation between differential methylation and gene expression levels. A probability value of *P* < 0.05 was considered statistically significant.

## Results

### Transcriptome

**Fig. 1:**
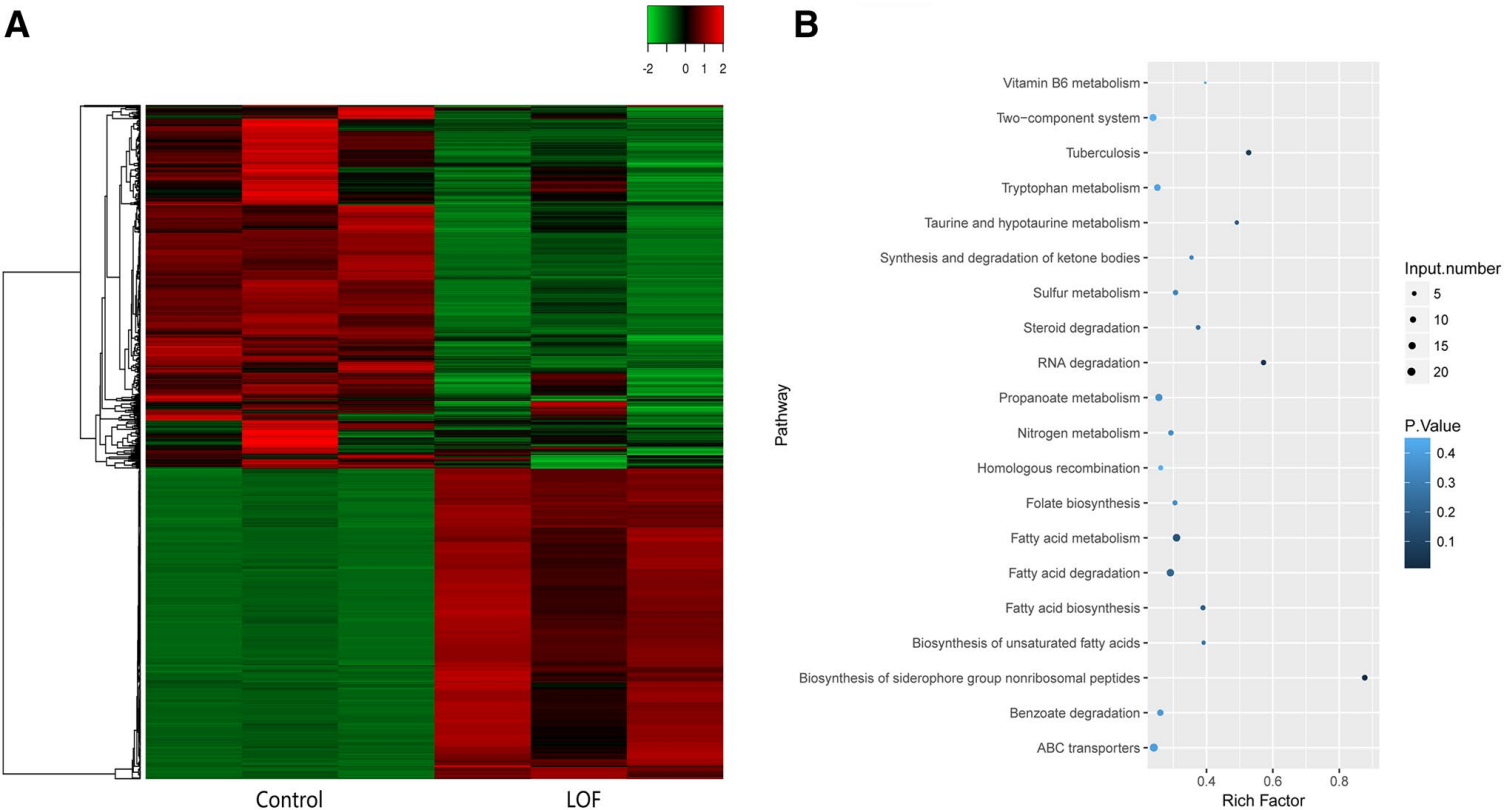
Transcriptome atlas and KEGG pathway analysis of DEGs between the LOF-resistant group and control group. **a** Heatmap of DEGs between the LOF-resistant group and control group. **b** KEGG pathway analysis of DEGs between the LOF-resistant group and control group. *DEGs* differentially expressed genes, *LOF* levofloxacin

In this study, the results suggested that 953 genes could be retrieved to be differentially expressed between the LOF-resistant and control groups (Fig. 1a and Table S1). Of these 953 DEGs, in the LOF-resistant group, 514 were significantly downregulated, and 439 were significantly upregulated. Moreover, all 953 DEGs were involved in 97 KEGG pathways and enriched in this study. In Fig. 1b and Table S2, enriched metabolic pathways (mtu01100) with 111 DEGs, biosynthesis of secondary metabolites (mtu01110) with 49 DEGs, and microbial metabolism in diverse environments (mtu01120) with 43 DEGs were presented; these pathways are significant to the further study of LOF resistance in *M. tuberculosis* H37Rv using transcriptome analysis.

### Methylome

**Fig. 2:**
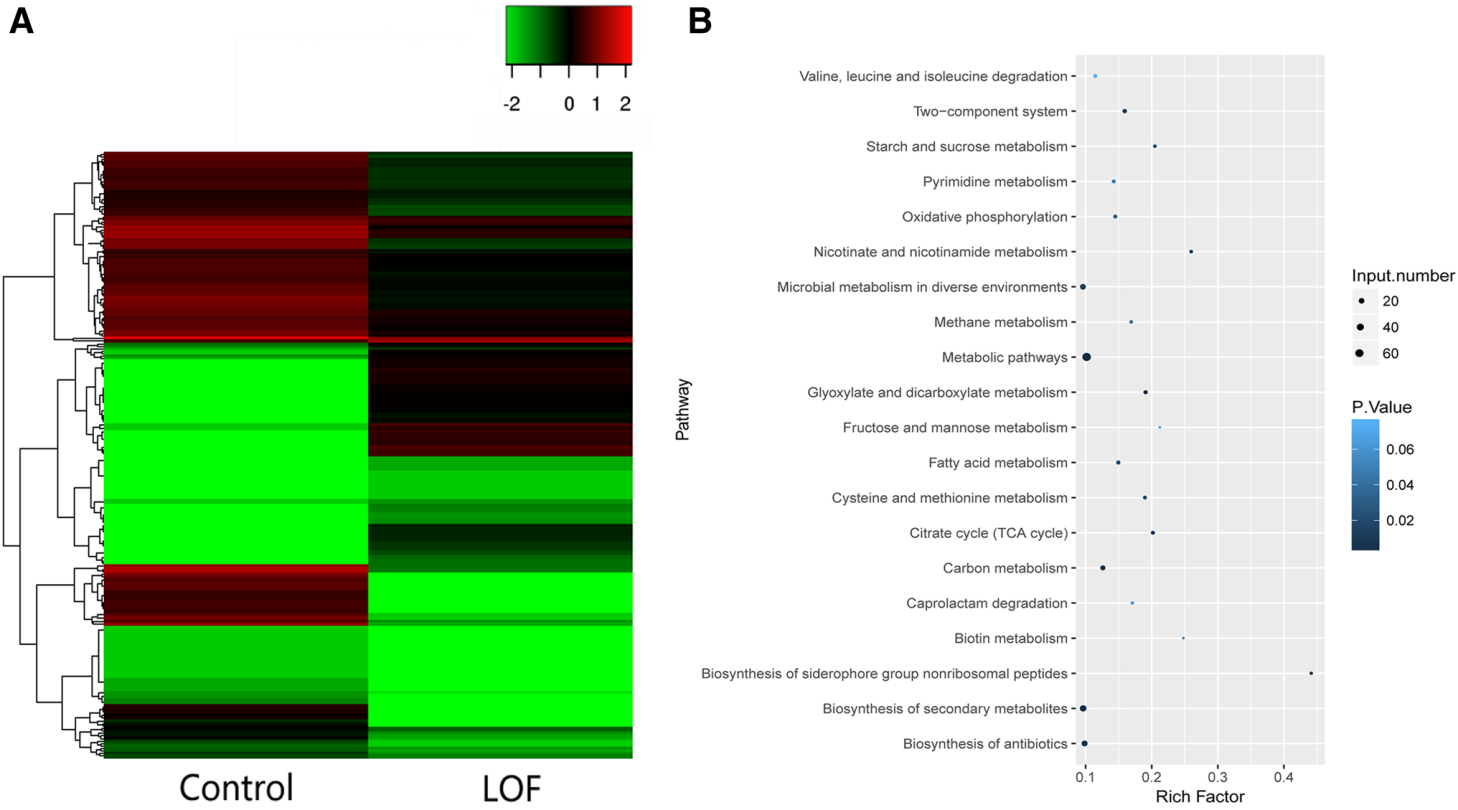
Methylome atlas and KEGG pathway analysis of DMGs between the LOF-resistant group and control group. **a** Heatmap of DMGs between the LOF-resistant group and control group. **b** KEGG pathway analysis of DMGs between the LOF-resistant group and control group. *DMGs* differentially methylated genes, *LOF* levofloxacin

In this study, 239 DMGs were identified (Table S3). In the LOF-resistant group, 150 DMGs were hypomethylated, and 89 DMGs were hypermethylated (Fig. 2a). Furthermore, the KEGG pathway analysis of these DMGs was conducted. The results indicate that all 239 DMGs were involved in 74 KEGG pathways (Fig. 2b and Table S4), including metabolic pathways (mtu01100) with 67 DMGs, biosynthesis of secondary metabolites (mtu01110) with 32 DMGs, and biosynthesis of antibiotics (mtu01130) with 25 DMGs; these pathways are significant to the further study of LOF resistance in *M. tuberculosis* H37Rv under methylome analysis.

### Overlap of Transcriptome and Methylome and Function Analysis

**Fig. 3:**
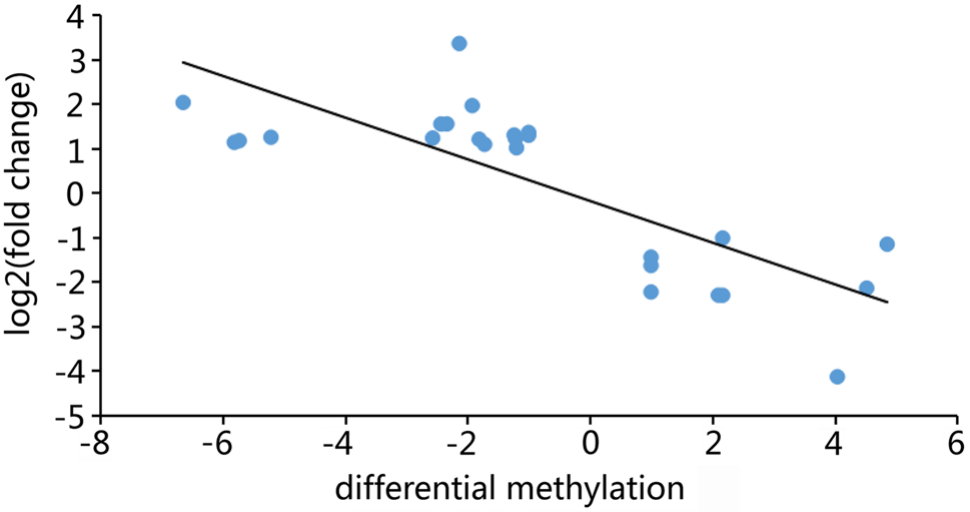
The correlation of gene expression levels and DNA methylation status. Scatterplots of gene expression fold change vs. DNA methylation change between the LOF-resistant group and control group. *LOF* levofloxacin

In order to obtain the overlapping genes of the DEGs and DMGs, we compared the two datasets. We obtained 25 overlapping genes (Table S5) after analyzing these two datasets. Of these 25 genes, 16 genes expressed upregulation and hypomethylation in the LOF-resistant group compared with those in the control group. Nine genes expressed downregulation and hypermethylation in the LOF-resistant group compared with those in the control group. These results were consistent with a previous study indicating that DNA hypomethylated and hypermethylated genes could promote and inhibit gene expression (Moore et al. 2013). In this study, we examined the associations among 25 overlapping genes between transcriptome and methylome. The results indicate that DMGs were negatively correlated with mRNA abundance (Fig. 3), which quantitatively verifies the negative relationship between methylation and gene expression in the LOF-resistant *M. tuberculosis* H37Rv strain.

**Fig. 4:**
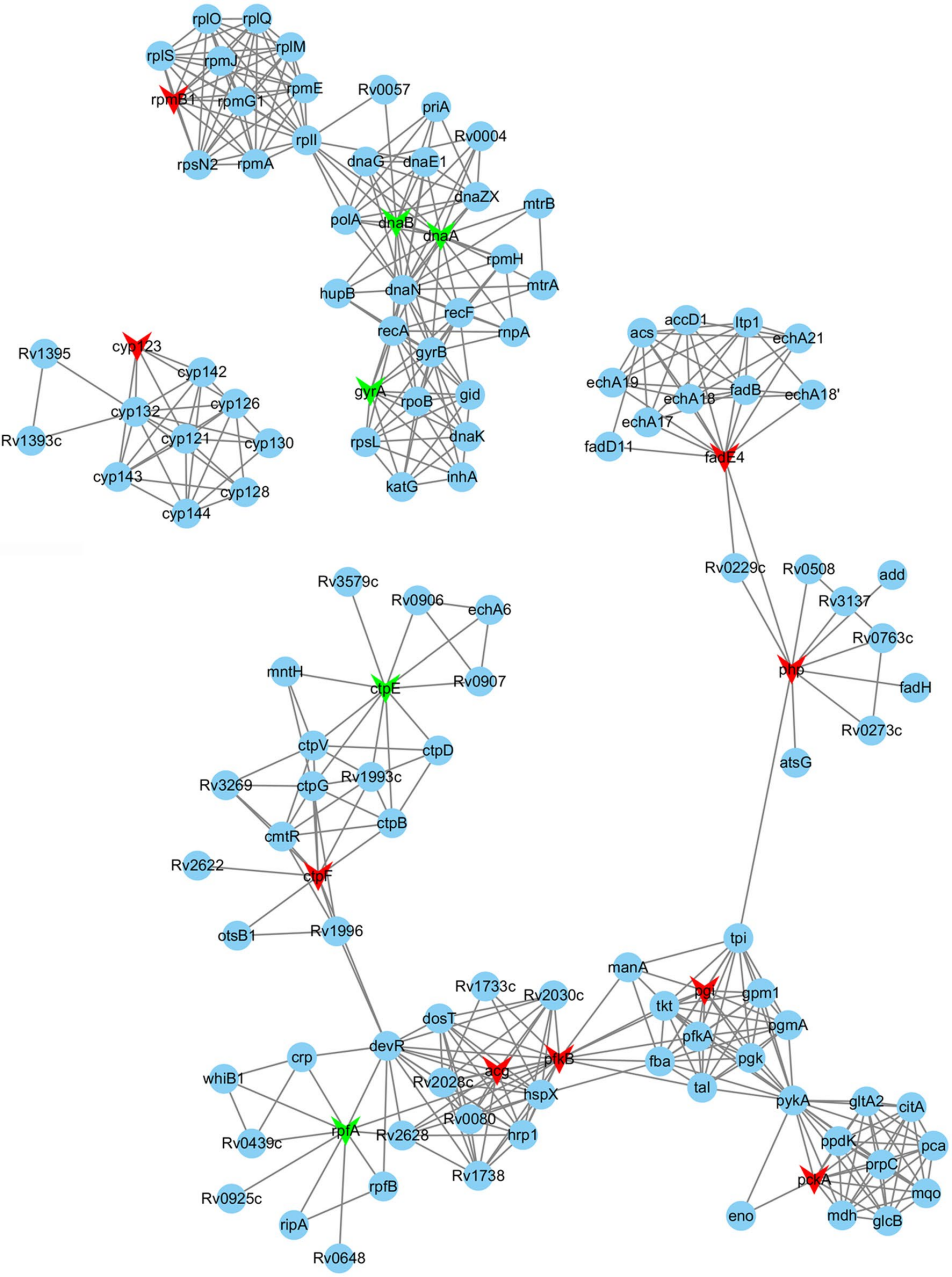
Analysis of protein–protein interaction networks of nine hypermethylated genes. The protein interaction networks for nine differentially methylated genes between the LOF-resistant group and control group. *LOF* levofloxacin; red, hypermethylated genes; green, hypomethylated genes; blue, other interacting proteins

In order to further study the functions of DMGs, all 25 genes were searched against the STRING database, as presented in Figure S1. It is notable that the hypermethylated genes *fadE4* (*Rv0231*), *php* (*Rv0230c*), *pgi* (*Rv0946c*), *pckA* (*Rv0211*), and *pfkB* (*Rv2029c*) interacted within various protein–protein interaction modules (Fig. 4). Additionally, these 25 genes were subjected to the KEGG pathway analysis, from which 25 signaling pathways were obtained (Table S6), including biosynthesis of antibiotics (mtu01130) with five genes and biosynthesis of secondary metabolites (mtu01110) with five genes; these pathways are significant to the further study of LOF resistance in *M. tuberculosis* H37Rv using a combination of transcriptome and methylome analysis. The result of protein–protein interaction (PPI) analysis would provide potential information regarding the associations of various *M. tuberculosis* proteins with levofloxacin resistance, which are valuable and warrant further research.

## Discussion

The treatment of MDR-TB is a difficult problem in current TB control. MDR-TB treatment has obvious disadvantages, such as high cost, low cure rate, and high mortality rate. Therefore, MDR-TB poses a significant threat to public health (Migliori et al. 2010). LOF has been proven effective in treating TB, which has been the subject of clinical trials to improve dosage amounts (Tiberi et al. 2018). According to the results obtained by Ghimire et al., there are several advantages of using LOF to treat MDR-TB. First, the concentration of LOF in tissues, such as sputum, bronchial mucosa, and lung tissue, could exceed that in the blood. Second, LOF could easily kill *M. tuberculosis* in macrophages. Third, continuous delivery of this drug has no accumulation tendency (Ghimire et al. 2016). Therefore, LOF could be an optimal drug for MDR-TB treatments and thus is in phase II trials (NTC01918397). However, the basic molecular mechanisms of LOF resistance in TB treatment have not been fully demonstrated.

In this study, we have studied transcriptome and methylome atlases of LOF-resistant *M. tuberculosis* H37Rv compared with the control group. The results suggested that 953 DEGs and 239 DMGs were obtained between the LOF-resistant and control groups. This result suggests that LOF resistance affects the change of internal regulation networks in *M. tuberculosis* H37Rv. Epigenetic abnormalities provide an alternative mechanism for transcriptional silencing. Therefore, we have focused on genes with downregulated mRNA expressions and upregulated methylation levels in both the LOF-resistant and control groups. Nine genes were identified: *pgi* (*Rv0946c*), *fadE4* (*Rv0231*), *php* (*Rv0230c*), *cyp132* (*Rv1394c*), *pckA* (*Rv0211*), *rpmB1* (*Rv0105c*), *pfkB* (*Rv2029c*), *acg* (*Rv2032*), and *ctpF* (*Rv1997*).

In *M. tuberculosis* H37Rv, *pgi* (*Rv0946c*) encodes a protein belonging to glucose-6-phosphate isomerase, which catalyzes the interconversion of d-glucopyranose 6-phosphate and β-d-fructofuranose 6-phosphate. This process is an essential step of the glycolysis and gluconeogenesis pathways. Apart from its enzymatic functions, *pgi* (*Rv0946c*) also has a number of other functions, including being an autocrine motility factor (AMF), a neuroleukin (NLK) agent, a serine proteinase inhibitor, and a differentiation and maturation mediator. As in most organisms, *pgi* (*Rv0946c*) is essential for the survival of *M. tuberculosis* in macrophages (Sassetti et al. 2003). Furthermore, a previous study demonstrated that *cyp132* (*Rv1394c*) plays a crucial role in *Rv1395*’s regulator function in cytochrome P450 regulation in *M. tuberculosis* (Recchi et al. 2003). Recchi et al. (2003) suggested that *Rv1395* could bind to two adjacent sites located between two divergent genes: a cytochrome P450 gene—*cyp132* (*Rv1394c*) and the *Rv1395* gene itself. *Rv1395* could induce the expression of the cytochrome P450 gene (*cyp132*) and repress its own transcription. Previous studies of metabolism in *M. tuberculosis* suggested that *pckA* (*Rv0211*) in *M. tuberculosis* can be related to anaplerotic and gluconeogenic routes. This suggests that *pckA* (*Rv0211*) might be related to the regulation of metabolic adaptations of central metabolism in various environments of *M. tuberculosis* (Beste et al. 2011; Watanabe et al. 2011). Moreover, as a member of the DOS regulator, the expression of *pfkB* (*Rv2029c*) is upregulated in hypoxia conditions and macrophages (Shi et al. 2016). The results of the mutation screening revealed that *pfkB* (*Rv2029c*) is not related to *M. tuberculosis* growth in vitro or in vivo (Phong et al. 2013; Sassetti and Rubin 2003). A previous study revealed that this gene encodes 6-phosphate fructokinase (*pfkB*), which is involved in the conversion of sugar 1-p into sugar 1,6-P (Phong et al. 2013). Recently, this protein was identified as a potential vaccine candidate (Zvi et al. 2008). Therefore, *pfkB* (*Rv2029c*) is potentially essential in vaccine development in *M. tuberculosis* treatment. Combined with the network of protein–protein interaction, we can also confirm that the hypermethylated genes *cyp132* (*Rv1394c*), *pckA* (*Rv0211*), and *pfkB* (*Rv2029c*) were essential in *M. tuberculosis* H37Rv with LOF resistance. The above genes were both enriched in biosynthesis of antibiotics (mtu01130) and biosynthesis of secondary metabolites (mtu01110) pathways in the LOF-resistant *M. tuberculosis* H37Rv group, which could be essential for the future study of *M. tuberculosis* treatment. In summary, multiple molecular mechanisms could facilitate in understanding the LOF-resistant mechanisms of *M. tuberculosis* H37Rv when mediated by methylation. Each of the genes identified in this study could be essential for future treatments of MDR-TB.

In this study, 953 genes were differentially expressed (LOF-resistant group/control group), metabolic pathways (mtu01100) was the most enriched in the KEGG pathway analysis. A total of 239 genes were differentially methylated (LOF-resistant group/control group), metabolic pathways (mtu01100) was also the most enriched in the KEGG pathway analysis. A total of 25 genes were overlapping between the transcriptome and methylome datasets, which may be the potential core genes of LOF function in *M. tuberculosis*. There were nine genes with downregulated mRNA expression and hypermethylation levels in the LOF-resistant group: *pgi*, *fadE4*, *php*, *cyp132*, *pckA*, *rpmB1*, *pfkB*, *acg*, and *ctpF*; in particular, *cyp132*, *pckA*, and *pfkB* deserve more in-depth study. The overlapping genes between transcriptome and methylome could be essential for studying the molecular mechanisms of LOF-resistant *M. tuberculosis* H37Rv. The above results could provide informative evidence for TB treatment using LOF.

## Electronic supplementary material

Below is the link to the electronic supplementary material.

Figure S1. The networks of protein–protein interactions for 25 overlapping differentially methylated genes between the LOF-resistant group and control group. *LOF* levofloxacin; red, hypermethylated genes; green, hypomethylated genes; blue, other interacted proteins. (TIF 2979 kb)

Table S1. Differentially expressed genes between the LOF-resistant group and control group. *LOF* levofloxacin. (XLS 178 kb)

Table S2. KEGG pathway analysis of differentially expressed genes between the LOF-resistant group and control group. *LOF* levofloxacin. (XLS 67 kb)

Table S3. Differentially methylated genes between the LOF-resistant group and control group. *LOF* levofloxacin. (XLS 41 kb)

Table S4. KEGG pathway analysis of differentially methylated genes between the LOF-resistant group and control group. *LOF* levofloxacin. (XLS 52 kb)

Table S5. Overlapping genes of differentially expressed genes and differentially methylated genes between the LOF-resistant group and control group. *LOF* levofloxacin. (XLS 24 kb)

Table S6. KEGG pathway analysis of overlapping genes between the LOF-resistant group and control group. *LOF* levofloxacin. (XLS 34 kb)

## Data Availability

Raw sequencing data presented in this paper are available under the GEO accession number PRJNA579441.
