# Supplementary figures and images for: Potential Genes Related to Levofloxacin Resistance in Mycobacterium tuberculosis Based on Transcriptome and Methylome Overlap Analysis
Source: J Mol Evol. 2020 Jan 9;88(2):202–9. doi: 10.1007/s00239-019-09926-z (PMC6989609; doi:10.1007/s00239-019-09926-z)

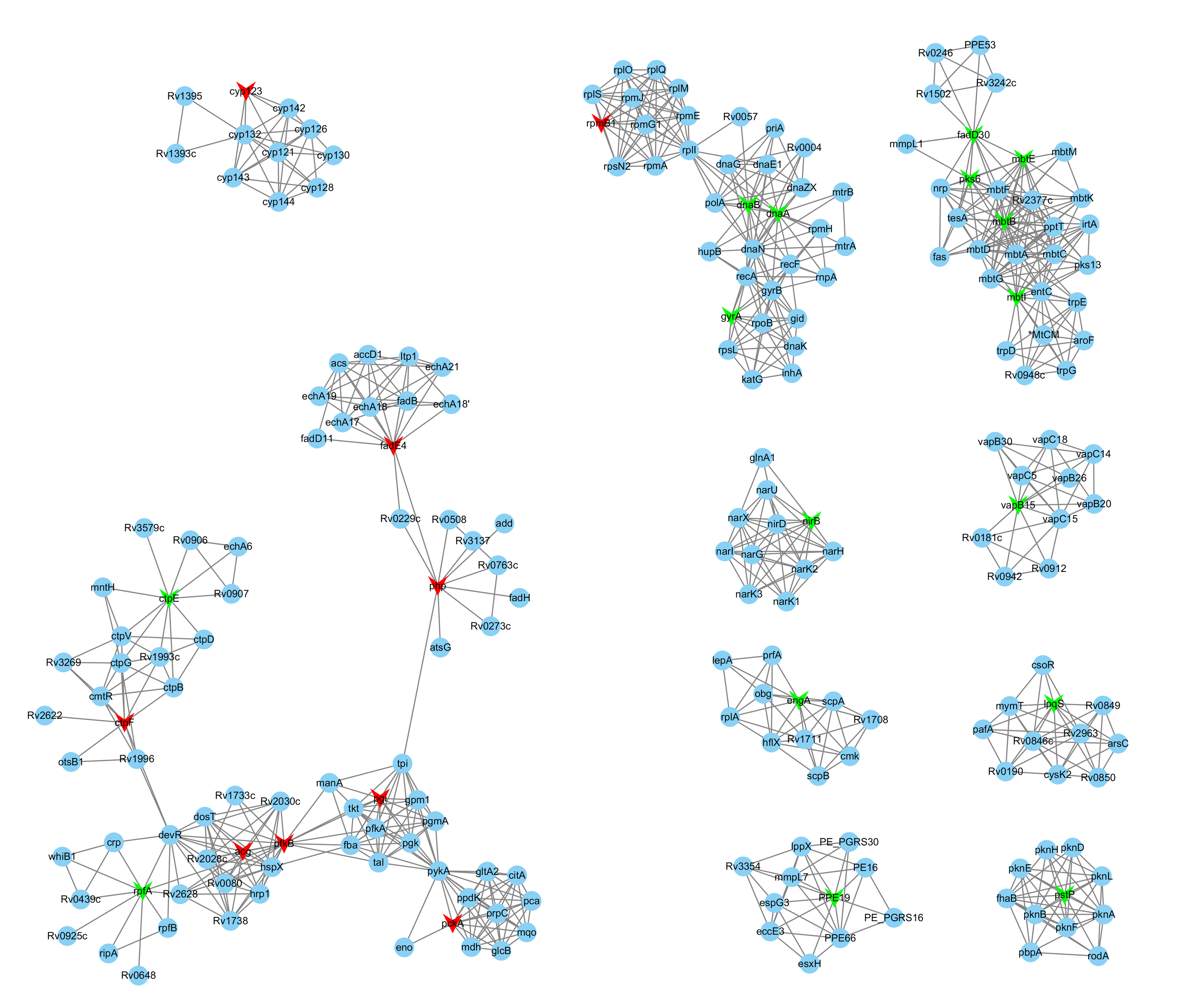

Supplement: Supplementary file 1 — Figure S1. The networks of protein–protein interactions for 25 overlapping differentially methylated genes between the LOF-resistant group and control group. LOF levofloxacin; red, hypermethylated genes; green, hypomethylated genes; blue, other interacted proteins. (TIF 2979 kb) [file 239_2019_9926_MOESM1_ESM.tif]
